# Utility of Ultrasound-Guided Attenuation Parameter (UGAP) in Renal Angiomyolipoma (AML): First Results

**DOI:** 10.3390/diagnostics14182002

**Published:** 2024-09-10

**Authors:** Paul Christian Kranert, Paula Kranert, Miriam C. Banas, Ernst Michael Jung, Bernhard Banas, Franz Josef Putz

**Affiliations:** 1 Department of Nephrology, University Hospital Regensburg, 93053 Regensburg, Germany; miriam.banas@ukr.de (M.C.B.); bernhard.banas@ukr.de (B.B.); franz-josef.putz@ukr.de (F.J.P.); 2 Department of Dermatology, University Hospital Regensburg, 93053 Regensburg, Germany; paula.kranert@ukr.de; 3 Department of Radiology, Interdisciplinary Ultrasound, University Hospital Regensburg, 93053 Regensburg, Germany; ernst-michael.jung@ukr.de

**Keywords:** UGAP, angiomyolipoma, tumour, renal imaging, examination conditions

## Abstract

Angiomyolipoma (AML) are the most common benign solid renal mass. Differentiation from malignant tumours is essential. Imaging features in ultrasound may overlap between malignant lesions, especially between renal cell carcinoma (RCC) and AML. So far, sectional imaging has been necessary for reliable differentiation. The aim of this study is to evaluate the use of the ultrasound-guided attenuation parameter (UGAP), a recently established tool for assessing hepatic steatosis, in the differentiation of AMLs from other renal masses. Therefore, 27 patients with unknown solid renal masses were examined by ultrasound including UGAP. The attenuation was assessed qualitatively by attenuation map and quantitatively in comparison to the surrounding renal tissue. UGAP was applicable in 26/27 patients. Findings were compared with CT/MRI as the current imaging standard. A total of 18 AML and 9 other renal tumours were found. The diagnostic performance of B-Mode (hyperechogenic lesion) ultrasound was 77.8% in identifying AML. The diagnostic performance of the attenuation map showed a diagnostic performance of 92.6%, whereby UGAP measurements were successful in 76.9% of cases. Quantitatively, we found a significant difference (*p* < 0.034) in mean measured attenuation between AML (0.764 ± 0.162 dB/cm/MHz) vs. other renal tumours (0.658 ± 0.155 dB/cm/MHz). The best performance was found by a combined parameter of a hyperechogenic lesion with a positive attenuation map with an accuracy of 95.0%. In conclusion, UGAP may represent a possibility for differentiating solid renal lesions more accurately by ultrasound, especially classic hyperechoic AMLs from other renal lesions. Further studies are needed to increase the diagnostic reliability further.

## 1. Introduction

Solid renal masses are frequent incidental findings on grey-scale ultrasound. Besides simple renal cysts, angiomyolipoma (AML) are the most frequent benign finding [1]. AML are the most common benign solid kidney tumour. They are typically composed of three types of tissue: dysmorphic blood vessels, smooth muscle, and fat. They mostly appear as sporadic cases (80%) but could also be linked to hereditary conditions (20%) like tuberous sclerosis and pulmonary lymphangioleiomyomatosis [1]. Sporadic AML are divided into several subtypes. The most common is the classic fatty type, which presents typically hyperechoic in grey-scale ultrasound [2]. Furthermore, in MRI, a distinction is made between other subtypes, such as the fat-poor, the hyperattenuating AML, the isoattenuation AML, the epithelioid AML, and the AML with epithelial cysts. AMLs are typically benign lesions, and treatment is mostly not necessary. However, AMLs of a distinct size have a relevant risk of spontaneous bleeding. There are a number of therapy options available for AML; however, there is no clear surveillance or discharge protocol, and, more crucially, no consensus or guidelines to handle these lesions [3]. A cut-off size for consideration of treatment has traditionally been 4 cm, based on a literature review and a case series from Oesterling et al. from 1986 [4]. According to more recent studies, bleeding risk only rises with larger tumours [5]. A recent literature review from Vaggers, Rice, et al. proposed a cut-off of 6–7 cm, with other factors such as the rate of growth, women in childbearing age, the size of the aneurysm, symptoms, and the risk of malignancy taken into account [6].

Essential for the further management of AMLs is the initial diagnosis and the differentiation from other hyperechoic, potential malign lesions. For diagnosis, a variety of imaging techniques are used, including ultrasound. Ultrasound screening is a non-invasive and widely available method. On grey-scale ultrasound, they may appear as single or multiple lesions which are primarily located in the renal cortex but can also grow into the renal sinus [7]. The echogenicity may vary due to the different ratio of fat, muscle, or vessels [8].

Although most AML are usually hyperechogenic, the current guidelines of the European Association of Urology (EAU) recommend further evaluation by computer tomography (CT) and magnetic resonance imaging (MRI) to differentiate between AML and other renal masses [9]. The American Urological Association guidelines for Evaluation, Management, and Follow up of renal masses and localized renal cancer also recommend a confirmation by pre/post contrast-enhanced CT or MRI for masses which were initially diagnosed by ultrasound imaging [10]. The diagnosis of AMLs in CT and MRI relies on the measurement of fatty tissue within the lesion. In CT, this is by carried out by measurement of Hounsfield units, which are typically 10 Hounsfield units (HU) or less for AML [11]. In MRI, differentiation is achieved by signal intensity in T1- and T2-weighted imaging [12].

Ultrasound-guided attenuation parameter (UGAP) is a recently established tool which is used for assessing hepatic steatosis [13]. Fat-rich tissue is characterized by a higher attenuation than other tissues.

Recent studies have approached differentiation of AML and RCC by CEUS. However, the characteristics of RCC and AML in CEUS remain still controversial. The description of enhancement pattern varies in different studies [14,15,16,17]. Although the analysis of CEUS quantitative parameters showed promising results, it has a low reproducibility and is very reliant on the operator’s expertise [18]. Reports have shown that approximately 5% of AML have low fat content [19]. UGAP has the potential to access the majority of AML with high fat content with a wide availability and reproducibility.

This retrospective study aimed to evaluate the diagnostic value of UGAP in the diagnosis of fat-rich AML in contrast to other renal masses. UGAP has the potential to improve the diagnostic accuracy of typical angiomyolipoma in ultrasound.

## 2. Methods

This single-centre study was conducted at the Department of Nephrology at the University Hospital Regensburg, Germany. Methodically, we retrospectively analysed prospectively collected data.

From May 2022 to January 2024, a total of 27 patients who had an ultrasound examination with detection of a focal solid renal mass and an additional UGAP measurement were included in the study. These patients were examined at the department of nephrology of the University Hospital of Regensburg. For these patients, ultrasound attained parameters, CT/MRI results, and pathology reports, if available, were obtained. The study was waived by the local ethics committee (17-662-101_P1, 17-662-101_P2, 17-662-101_P3).

Ultrasound was executed independently by an experienced nephrologist during routine ultrasound examinations in the Department of Nephrology of the University Hospital of Regensburg. The investigations were performed using a LOGIQ E10 ultrasound system (GE Healthcare, Wauwatosa, WI, USA) with a C1-6-D convex array probe on patients in a supine position with the arm in abduction.

If a focal renal lesion was detected during the examination, size, location, depth, echogenicity homogeneity, and vascularization in colour-coded Doppler technique were documented. Afterward, the UGAP measurements were carried out.

The UGAP measurement is based on a tissue-mimicking reference phantom with a known attenuation coefficient [14]. Due to this model, imaging conditions such as frequency (3.5 MHz) and depth of the ROI (4–8 cm) are fixed due to the manufacturer’s requirements. The ROI can be moved horizontally. Based on the attenuation rate, a color-coded local attenuation value is displayed for each pixel within the measurement box. Due to the composition of the fat-rich AML, high amounts of fat tissue lead to higher attenuation. The colour scale runs from red for intense attenuation (high content of fat) to blue for low attenuation (low content of fat).

**Figure 1:**
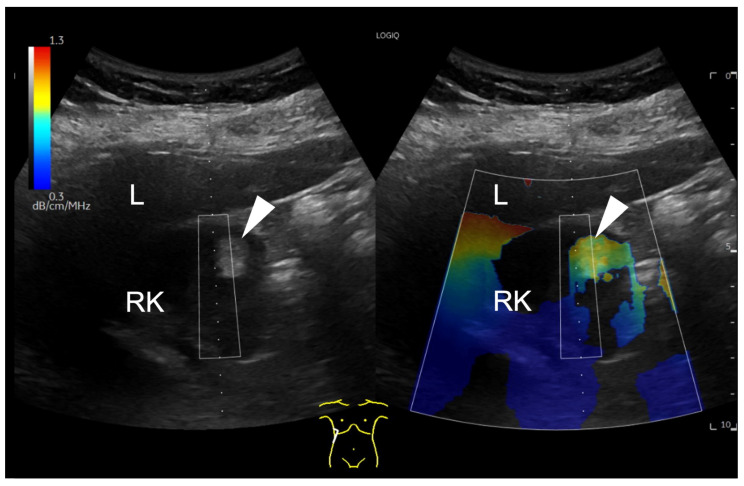
B-mode ultrasound image showing a focal hyperechoic lesion (arrowhead) in the cortex of the right kidney (RK). In the attenuation map on the right side, the lesion appears in red and yellow, representing a higher attenuation than the surrounding tissue. The level of attenuation is shown color-coded on the scale on the top left corner of the image. Next to the right kidney, the liver (L) is displayed. A region of interest (ROI) box is displayed above the hyperechogenic lesion.

First, UGAP was used qualitatively. Using the quality map option, the color-coded map was placed right above the lesion. A positive finding was defined by a significant difference in the colour map above the lesion (red-yellow) compared to the surrounding tissue. This is shown for example in Figure 1.

Secondly, UGAP was used quantitatively. Depending on the size, up to three measurements were taken in the region of interest (ROI). In addition, up to three more measurements were performed in the renal cortex close to the lesion for comparison.

Methodically, the placement of the ROI was chosen in such way that the lesion to be assessed was captured as completely as possible. Furthermore, the remaining part of the ROI should capture as much kidney tissue as possible as a comparison. The proportion of surrounding tissue other than renal cortex should be kept as small as possible to avoid distortion.

**Figure 2:**
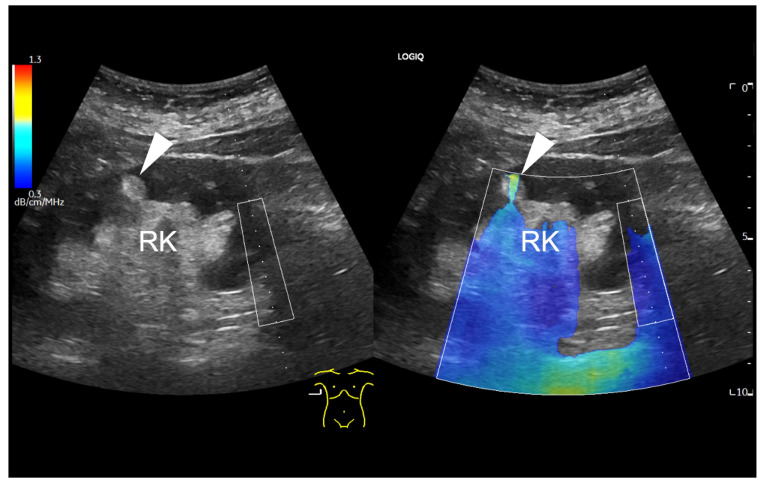
B-mode ultrasound of the right kidney (RK), displaying a focal hyperechogenic lesion (arrowhead) in the cortex, which is barely included in the colour coded attenuation map. The ROI box on the right side of the map can be only moved horizontally due to methodological reasons. Due to the superficial localization of the small renal lesion, the attenuation colour map is only partial applicable (arrowhead).

Findings in which the measurement was influenced by given factors, findings without the possibility of a measurement, and findings with no possible measurement but with a positive, fat-rich visual finding in the colour map were recorded in the database separately. An example of a focal renal lesion that was not accessible for UGAP measurement is shown in Figure 2.

All lesions were examined by CT or MRI. The diagnosis of an AML was made based on the presence of a high fat content in the lesion. Therefore, in the CT scan, the fat content of the lesion was measured within the unenhanced, naïve scan. The Hounsfield units were correlated with the results of the UGAP measurements. In MRI, the fat content was estimated by the typical signal within the fat saturated sequences. These results from cross-sectional imaging contributed to the current imaging standard for the performance analysis of UGAP.

In the case of a potential malignant lesion, the patient was admitted to the urologists and the histological results were also included to the analysis.

The statistical analysis was performed using SPSS statistical software (IBM SPSS Statistics 29.0; Chicago, IL, USA) and Microsoft Excel^®^ 2016 (Redmond, WA, USA). Cross tables were used to define the sensitivity, specificity, positive predictive value (PPV), negative predictive value (NPV), and diagnostic accuracy.

## 3. Results

We included 27 patients (10 male, 17 female) who underwent ultrasound examination in the Department of Nephrology of the University Hospital of Regensburg. The age at the time of the examination was between 22 and 82 (mean 62.11 ± 14.01 years).

In 20 patients (74.1%), an AML was presumed according to the ultrasound findings. The lesions were typical hyperechogenic in 74.1% (*n* = 20), isoechogenic in 18.5% (*n* = 5), and hypoechogenic in 7.4% (*n* = 2) of all cases. Typical imaging features of AML in different modalities are described in Table 1.

The mean diameter of the lesions was 2.16 ± 1.43 cm, and the mean depth was 4.57 ± 1.12 cm. Vascularisation at the margins was displayed by colour Doppler mode in 70.4% (n = 19); in 29.6% (n = 8) of cases, the lesion showed no vascularization.

Seven lesions (25.9%) were located at the upper renal third, seven lesions (25.9%) at the lower third, and thirteen lesions (48.1%) in between. Eighteen lesions (66.7%) were found in the right kidney, seven lesions (25.9%) in the left kidney, and twice (7.4%) a lesion was found in a kidney transplant. In 16 cases (59.3%), the lesion was homogeneous, while in 11 cases (40.7%), it was inhomogeneous.

The UGAP measurement was technically possible in 26 of 27 cases (96.3%). Technical limitations (e.g., only partial colour coding of the lesion) appeared in five cases (18.5%). In one case (3.7%), no measurement was viable due to the depth of the lesion.

The attenuation-map displayed a high attenuation rate in 20 cases (74.1%), while in 7 cases (25.9%), a low attenuation was displayed. The result assumed from the attenuation-map corresponded in 25 cases (92.59%) with the result of the cross-sectional imaging. The sensitivity and specificity of the attenuation map is 100%/77.8% (PPV 90%, NPV 100%, diagnostic accuracy 92.6%).

**Figure 3:**
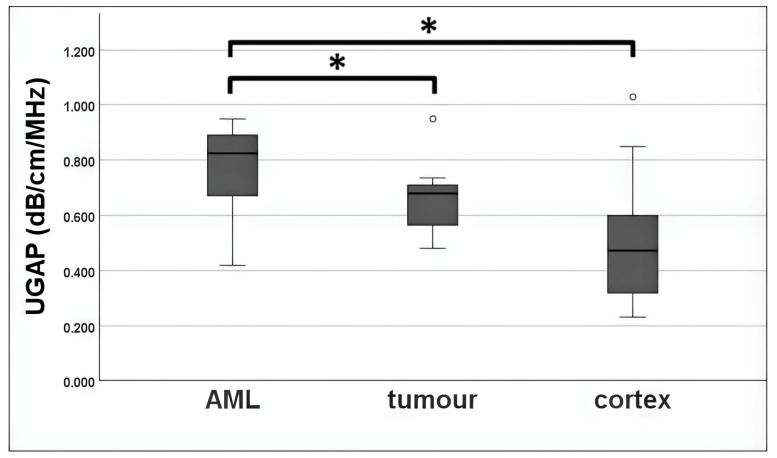
Boxplot of UGAP measurement in comparison for AML, tumour and cortex. Mean measured UGAP values were 0.780 ± 0.157 dB/cm/MHz for the AML subgroup, 0.666 ± 0.135 dB/cm/MHz for the tumour subgroup, and 0.498 ± 0.194 dB/cm/MHz for cortex. Outliers are displayed by dots. Mean attenuation of AML group differed significantly from tumour group (*p* = 0.034) and cortex (*p* < 0.001), which is displayed by the symbol *. No significant differences were detectable between tumour and renal cortex (*p* = 0.093).

UGAP measurement was possible in 96.3% of all cases. The mean UGAP values of the AML subgroup (0.780 ± 0.157 dB/cm/MHz) were higher than those of the tumour subgroup (0.666 ± 0.135 dB/cm/MHz). A mean value was also calculated from the three measurements in the cortex next to the lesion. The mean measured attenuation in the cortex was 0.498 ± 0.194 dB/cm/MHz overall. There were statistically significant differences between AML vs. tumour (*p* = 0.034) and AML vs. cortex (*p* < 0.001). No significance was detectable between tumour and cortex (*p* = 0.093). A boxplot is given in Figure 3.

The cut-off value for a positive diagnostic result by UGAP measurement was calculated by an ROC analysis based on Youden’s index. Based on the calculation, lesions with a higher attenuation than the calculated cut-off of 0.745 dB/cm/MHz were defined as positive for UGAP measurements and were considered to be an AML.

In all cases, imaging data was obtained. CT data was available in 21 cases, MRI data was available in 10 cases, and in 4 cases, both modalities were performed.

CT-Scan found an AML in 13 cases, and in 8 cases, a renal tumour was suspected, while in MRI, an AML was displayed in 8 cases, and in 2 cases, a renal tumour was found. Combining the results of both imaging modalities, in total, 18 AML were found, and 9 lesions were described as renal tumour. These subdivided into two papillary renal cell carcinoma, one clear cell renal cell carcinoma, one oncocytoma, one renal metastasis, one renal lymphoma, one renal capsular thickening, and two renal lesions which did not correspond to an AML on cross-sectional imaging and are under further investigation.

The pooled data of CT and MRI as a current imaging standard for UGAP showed a sensitivity and specificity of 70.6%/88.9% (PPV 92.3%, NPV 61.5%, accuracy 76.9%) for UGAP measurement. Taking the visual results from the colour map into account, a sensitivity and specificity of 100%/77.8% (PPV 90%, NPV 100%, accuracy 92.6%) was shown. A cross table is given in Table 2.

**Figure 4:**
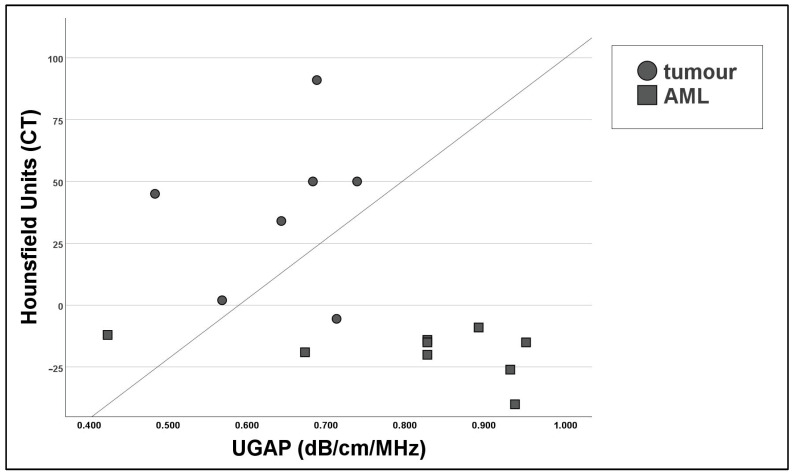
Scatter plot to compare UGAP measurements and Hounsfield units from unenhanced CT scans, showing a negative non-linear relationship. All results are statistically significant (Pearson’s correlation test *p* < 0.001).

The mean HU (Hounsfield units) measured in the ROI was −23.00 ± 15.69 in the AML group and 38.07 ± 32.53 in the other renal masses. The relationship between the Hounsfield measurements in CT and UGAP measurements was determined through Pearson’s correlation coefficient, which showed a strong negative correlation (*r* = −0.649) which was significant (*p* = 0.007). A scatter plot is shown in Figure 4.

To improve diagnostic performance, we combined the criteria of hyperechoic lesion in B-Mode ultrasound, (defined in correlation to the renal cortex) with a positive visual finding in the colour map (fat-rich). In 16 cases (61.5%), both conditions were met. Compared with the results of the cross-sectional imaging, the combined parameter showed a sensitivity of 100% and a specificity of 75% (PPV 94.1%, NPV 100%, accuracy 95.0%). The overview of the diagnostic performance of the different methods is given in Table 3. A correlation between the combined parameter and the CSI outcome was tested through Pearson’s correlation coefficient, showing a positive correlation (*r* = 0.730) which was significant (*p* < 0.001). In this subgroup, a measurement was possible in 15 cases (93.8%).

## 4. Discussion

Renal masses are often found incidentally in abdominal imaging. AML are the most common benign solid renal lesion. In grey-scale B-Mode ultrasound, the common fatty type of AML presents as a focal hyperechoic lesion [21], while other subtypes may differ. Differentiation between AML and other malign lesions is crucial. For a reliable diagnosis, besides ultrasound, there is the need for further cross-sectional imaging as the current imaging standard. The evidence of macroscopic fat within the lesion is the diagnostic criterium in all modalities to affirm the diagnosis of an AML. The current imaging standard to determine the fat content is CT or MRI. Due to its wide availability and relatively low costs, CT is commonly used. For example, in the unenhanced CT, the AML presents as a hypodense mass due to its fat content. Hounsfield units (HU) are a unit used to measure the attenuation of X-rays in tissue. For AML, the commonly used attenuation threshold in literature is below -10 HU on unenhanced CT.

UGAP is a recently established tool used to access hepatic steatosis in ultrasound. In suitable high-end ultrasound machines, this technique could be applied easily without further equipment using the standard ultrasound probe. Ultrasound beams attenuate during the transition between organs due to interaction with the tissue and tissue boundaries. Due to diffusion, scattering, and absorption, the strength of the signal is weakened. The measurement is based on a tissue-mimicking reference phantom with a known attenuation coefficient [22]. The use of UGAP in other tissues besides the liver is not common and has not been evaluated so far.

The aim of this study was to evaluate the use of UGAP in the kidney and to analyse the diagnostic value of UGAP in the differentiation of AML and other solid renal mases in comparison to cross-sectional imaging (CT or MRI) as the current imaging standard.

There are two possible ways to use UGAP. At first, UGAP could be used to show the fat content of different tissues in a colour map. Secondly, the fat content could be quantified within a region of interest. In our study, UGAP measurements on the kidney were technical successful in 26 out of 27 examinations (96.3%). The colour map was more stable and could be retrieved in more cases than the UGAP quantification. The colour map was even applicable in small lesions up to 0.7 cm in diameter. The diagnostic accuracy in the identification of an AML was 92.6%.

The attenuation of solid renal lesions was possible in 26 out of 27 lesions (96.3%). A cut-off value for AMLs could be established at 0.745 dB/cm/MHz. This allows a diagnostic accuracy of 76.9% for the diagnosis of AMLs. The mean measured attenuation values of the solid lesions correlate with the results of the Hounsfield measurements in the correlating CT scan (*p* < 0.001) (Figure 4).

In the subgroup of hyperechoic lesions, the diagnostic performance of UGAP is even higher (sensitivity of 100%, specificity of 75% PPV 94.1%, NPV 100%, diagnostic accuracy 95%).

**Figure 5:**
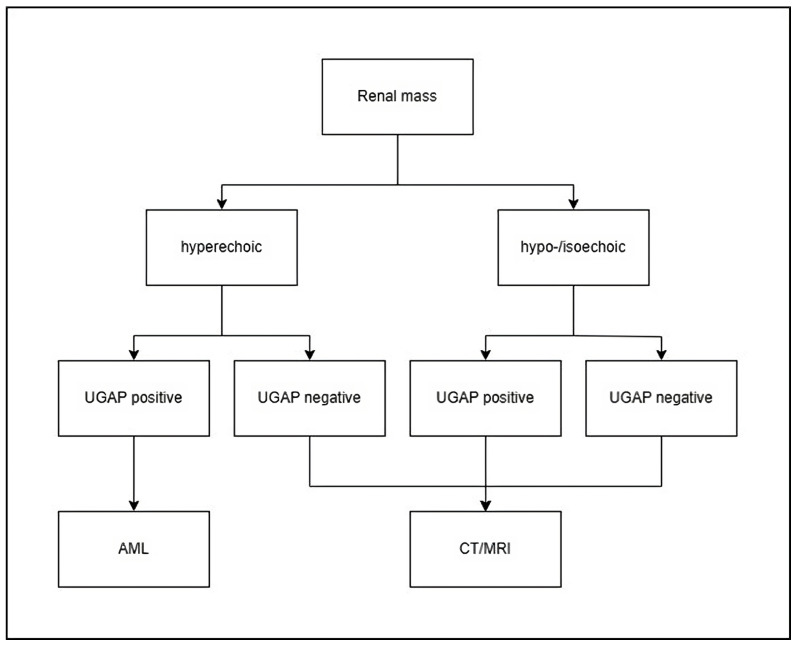
Proposal for a potential algorithm using UGAP for focal renal lesions. The algorithm was developed by the authors based on the findings of this study.

With respect to the good diagnostic accuracy and the high NPV, a possible diagnostic algorithm could be proposed (Figure 5).

Therefore, we suggest that a positive attenuation signal in the colour map of UGAP in a solid hyperechoic renal lesion could be used as a new diagnostic criterion for AMLs, without the need for further imaging, e.g., CT or MRI.

The use of this new method allows us to increase the diagnostic accuracy of detecting an AML by about 20% in comparison to grey-scale ultrasound. Furthermore, for this method, there is no contrast agent necessary.

Our study has some limitations: In its current state, the UGAP measurement is limited to a defined field of depth and frequency; therefore, not all lesions may be accessible. Especially when the lesion is located next to the edge of the ROI, a measurement may be distorted. In our study, not all renal lesions could be captured in their entirety (five cases, 19.2%). Colour maps could be retrieved in more cases than the quantification of the fat attenuation. Especially when lesions were located at the concave side of the kidney, they were not accessible for attenuation measurements, although a signal in the color-coded map could be seen. Improvements in the technique may overcome this problem in future.

Measurements may be affected due to the surrounding inhomogeneous tissue around the relevant renal lesion. In particular, the assessment of exophytically growing masses may be distorted. In these cases, further imaging is required to differentiate the lesion. A limitation for these initial results is the small sample size of 27 patients. Due to the short-term availability of the UGAP measurement, the number of examined renal lesions was limited. With a standardized implementation of the UGAP measurement in the examination of focal renal lesions, higher patient numbers can be achieved in the future. Further studies are needed to increase the diagnostic reliability even further, especially including non-typical AML subtypes.

## 5. Conclusions and Future Works

AML are the most common benign solid renal lesions. UGAP represents a new possibility to differentiate solid renal lesions more accurately. This may be accessed by both UGAP measurement and visually by colour map. Our results demonstrated that there is an increase in attenuation in AMLs compared to other renal masses and the surrounding renal cortex. For a hyperechoic lesion with a positive finding in the colour map, a good sensitivity and specificity could be shown. Based on these initial results, in the case of a hyperechoic kidney lesion with a clear positive signal in colour mapping and significantly increased attenuation in the UGAP measurement, further cross-sectional imaging diagnostics could be dispensed. All other kidney lesions should be clarified additionally according to the current imaging standard by CT and MRI. UGAP has the potential to be a widely available, non-invasive option for the differentiation of focal hyperechogenic renal lesions without the need for cross-sectional imaging, avoiding unnecessary examinations, radiation exposure, or invasive procedures. In the next step, we plan to increase the number of examined renal lesions due to a standardized implementation of UGAP measurement for all kind of focal renal lesions. The aim is to further increase diagnostic accuracy and collect qualitative and quantitative data for all other types of renal masses. The other subtypes of renal angiomyolipoma, e.g., low-fat AML, are of particular interest.

## Figures and Tables

**Table 1:** Imaging features of angiomyolipoma (AML).

AML Type	Ultrasound	CT	MRI
classic	hyperechoic	fat attenuation (-10 HU)	T2-hyperintense, reduction in signal intensity in CSI
other subtypes	iso to hypoechoic	iso to hyperattenuating	T2-hypointense

Typical characteristics of AML types in different diagnostic modalities. Table modified from Jinzaki et al. [20].

**Table 2:** Cross table of the current imaging standard cross-sectional imaging (CSI, e.g., CT/MRI) vs. UGAP results.

A.		
UGAP Measurement Result	CSI Result	
	AML	tumour
AML	12	1
tumour	5	8
total	17	9
**B.**		
**UGAP Colour Map**	**CSI Result**	
	AML	tumour
AML	18	2
tumour	0	7
total	18	9
**C.**		
**Echogenicity in B-Mode Ultrasound**	**CSI Result**	
	AML	tumour
hyperechoic	16	4
iso-/hypoechoic	2	5
total	18	9
**D.**		
**Combined Parameter of Echogenicity and UGAP Colour Map**	**CSI Result**	
	AML	tumour
AML	16	1
tumour	0	3
total	16	4

Cross table of the UGAP measurement results vs. the current imaging standard (CT/MRI). A: UGAP results in comparison with a combined parameter of the result of any cross-sectional imaging (CSI). B: UGAP colour map in comparison with a combined parameter of the result of any cross-sectional imaging. C: Echogenicity in B-mode ultrasound in comparison with a combined parameter of the result of any cross-sectional imaging. D: Combined parameter of hyperechoic echogenicity and high attenuation signal in UGAP colour map in comparison with a combined parameter of the result of any cross-sectional imaging.

**Table 3:** Overview of the diagnostic performance of the different ultrasound methods in comparison to the current imaging standard (CT/MRI).

	Sensitivity	Specificity	PPV	NPV	Accuracy
UGAP measurement	70.6%	88.9%	92.3%	61.5%	76.9%
UGAP colour map	100%	77.8%	90.0%	100%	92.6%
hyperechoic	88.9%	55.6%	80.0%	71.4%	77.8%
hyperechoic + UGAP colour map	100%	75.0%	94.1%	100%	95.0%

Tabular overview of the different modalities in comparison with the result of cross-sectional imaging (CSI, e.g., CT or MRI) as current imaging standard.

## Data Availability

The data that support the findings of this study are available on request from the corresponding author. The data are not publicly available due to privacy and the data policy of the University.
